# Supplementary material for: Genome-Wide Identification of Seven in Absentia E3 Ubiquitin Ligase Gene Family and Expression Profiles in Response to Different Hormones in Uncaria rhynchophylla
Source: Int J Mol Sci. 2024 Jul 11;25(14):7636. doi: 10.3390/ijms25147636 (PMC11277444; doi:10.3390/ijms25147636)
Supplement: Supplementary file 1 [file ijms-25-07636-s001.zip › Table S1.pdf]

Table S1 The primers used for expression analysis

Gene_name	Forward primer (5'-3')	Reverse primer (5'-3')
UrSINA1	CTGAATACGAAGACGAAGAC	GGCTGATGGTGATGATTG
UrSINA2	AGAGTGGCTGAATCATTAGA	ACTGTATGGACGGAATCG
UrSINA3	CGGTCTTCAACTGTTATGG	TATGGCTGTCACGGATAC
UrSINA4	GGTAGCACAGTTGGTAGA	CCAGTTATAGGACATTCATCTC
UrSINA5	AAGTGGCAGAGTCATTAGA	GAGCAACAAGGAATTGGATA
UrSINA6	AAGTGGCAGAGTCATTAGA	GAGCAACAAGGAATTGGATA
UrSINA7	TACTCGGCAATGTCAACT	GGAGCACTGATGAATAGGA
UrSINA8	CCACTACTTCTGCCTACAT	TTCTGCTGTTCCTTCCAT
UrSINA9	GCATCAATACGCTTCTTCA	TACAGGTGGAACAGAGTG
UrSINA10	CACTTGGATGCTGACTGT	ACCGACTTCTAGGCTGTA
UrGES	*AGACACAGAAGGATGGCAAGG*	*ACCACCGAGTAATCTCAGACAAC*
UrG10H	*GCTGAGGTGACTGAACTATTAGG*	*GTGTCCGCTGGTCAATTATCT*
Ur10HGO	*CTCTGCTGTTCATCCTCTCCTT*	*CCAATGCCACTTCCACCTAATATC*
UrIO	*GCTGGTCTTCTTCTCCTTCTGATAA*	*ACTGGTCCGTACTTGCTTCTC*
Ur7DLGT	*CCGCAACAGAGCAGTCAATG*	*CCTCATCACCCACAGAAACCT*
Ur7DLH	*TGCCGACGAAGAGGAACAA*	*ATGCCCAAGAAGTTATCACCAGTA*
UrLAMT	*AGGAGATTCAGGACAAGA*	*TTACGAGTTCCATCAAGG*
UrSLS	*CTCATAGGTGCATTGATGTGGTTG*	*ACTGGTCCGTACTTGCTTCTC*
UrAS	*CGGAATCAATCGGTTGGAGTCT*	*AACACTGCCTTCTCGTAGTCTTC*
UrTDC	*CGTTACAAACTGAACCCAAACCAG*	*GGTTTCTTGTGGAAGATTTCCACCTC*
UrSTR	*CCATCTTCGCTAAGAAATCAAACCAAA*	*GTTGCTGATTTTTCCTCACTTTTTGGG*
UrSGD	*GATACACAAGAGGACAGAGA*	*GGAAGGTTGGCTACACTA*
