# Supplementary material for: Genome-Wide Identification of Seven in Absentia E3 Ubiquitin Ligase Gene Family and Expression Profiles in Response to Different Hormones in Uncaria rhynchophylla
Source: Int J Mol Sci. 2024 Jul 11;25(14):7636. doi: 10.3390/ijms25147636 (PMC11277444; doi:10.3390/ijms25147636)
Supplement: Supplementary file 1 [file ijms-25-07636-s001.zip › Table S2.pdf]

Table S2 The syntenic analysis of *SINA* genes between *U. rhynchophylla* and *A.thaliana,* and *C. camephora*

Gene_ID	Ur_Chrom	Gene_ID	Chrom	Species
g6840.t1	chr2	transcript:AT1G66620.1	chr1	*Arabidopsis thaliana*
g6840.t1	chr2	transcript:AT5G37870.1	chr5	*Arabidopsis thaliana*
g29173.t1	chr4	transcript:AT2G41980.1	chr2	*Arabidopsis thaliana*
g29173.t1	chr4	transcript:AT3G58040.3	chr3	*Arabidopsis thaliana*
g21951.t1	chr5	transcript:AT2G41980.1	chr2	*Arabidopsis thaliana*
g21951.t1	chr5	transcript:AT3G58040.3	chr3	*Arabidopsis thaliana*
g4133.t1	chr16	transcript:AT3G61790.1	chr3	*Arabidopsis thaliana*
g4133.t1	chr16	transcript:AT4G27880.1	chr4	*Arabidopsis thaliana*
g41269.t1	chr18	transcript:AT4G27880.1	chr4	*Arabidopsis thaliana*
g41269.t1	chr18	transcript:AT5G53360.1	chr5	*Arabidopsis thaliana*
g6840.t1	chr2	gene-GSCOC_T00040195001	HG974434.1	*Coffee canephora*
g29173.t1	chr4	gene-GSCOC_T00030791001	HG974433.1	*Coffee canephora*
g21951.t1	chr5	gene-GSCOC_T00030791001	HG974433.1	*Coffee canephora*
g29806.t1	chr7	gene-GSCOC_T00013207001	HG974428.1	*Coffee canephora*
g29806.t1	chr7	gene-GSCOC_T00019257001	HG974428.1	*Coffee canephora*
g41916.t1	chr8	rna-GSCOC_T00038020001	HG974428.1	*Coffee canephora*
g26498.t1	chr8	gene-GSCOC_T00013207001	HG974428.1	*Coffee canephora*
g4133.t1	chr16	gene-GSCOC_T00019981001	HG974429.1	*Coffee canephora*
g4133.t1	chr16	gene-GSCOC_T00027716001	HG974429.1	*Coffee canephora*
g41269.t1	chr18	gene-GSCOC_T00019981001	HG974429.1	*Coffee canephora*
g41269.t1	chr18	gene-GSCOC_T00027716001	HG974429.1	*Coffee canephora*
